# Urinary mRNA Expression of Glomerular Podocyte Markers in Glomerular Disease and Renal Transplant

**DOI:** 10.3390/diagnostics11081499

**Published:** 2021-08-20

**Authors:** Silvia Armelloni, Deborah Mattinzoli, Masami Ikehata, Carlo Alfieri, Mirco Belingheri, Gabrilella Moroni, Donata Cresseri, Patrizia Passerini, Roberta Cerutti, Piergiorgio Messa

**Affiliations:** 1 Renal Research Laboratory, Fondazione IRCCS Cà Granda Ospedale Maggiore Policlinico, 20122 Milan, Italy; silvia.armelloni@policlinico.mi.it (S.A.); deborah.mattinzoli@policlinico.mi.it (D.M.); masami.ikehata@policlinico.mi.it (M.I.); 2 Department of Clinical Sciences and Community Health, University of Milan, 20122 Milan, Italy; piergiorgio.messa@unimi.it; 3 Department of Nephrology, Dialysis and Renal Transplantation, Fondazione IRCCS Cà Granda Ospedale Maggiore Policlinico, 20122 Milan, Italy; mirco.belingheri@policlinico.mi.it (M.B.); gabriella.moroni@policlinico.mi.it (G.M.); donata.cresseri@policlinico.mi.it (D.C.); patrizia.passerini@policlinico.mi.it (P.P.); roberta.cerutti@policlinico.mi.it (R.C.)

**Keywords:** renal transplantation, glomerular disease, urine biomarkers, podocyte mRNA, signaling molecules

## Abstract

The research of novel markers in urinary samples, for the description of renal damage, is of high interest, and several works demonstrated the value of urinary mRNA quantification for the search of events related to renal disease or affecting the outcome of transplant kidneys. In the present pilot study, a comparison of the urine mRNA expression of specific podocyte markers among patients who had undergone clinical indication to renal transplanted (RTx, *n* = 20) and native (N, *n* = 18) renal biopsy was performed. The aim of this work was to identify genes involved in podocytes signaling and cytoskeletal regulation (*NPHS1, NPHS2, SYNPO, WT1, TRPC6, GRM1*, and *NEUROD*) in respect to glomerular pathology. We considered some genes relevant for podocytes signaling and for the function of the glomerular filter applying an alternative normalization approach. Our results demonstrate the WT1 urinary mRNA increases in both groups and it is helpful for podocyte normalization. Furthermore, an increase in the expression of *TRPC6* after all kinds of normalizations was observed. According to our data, WT1 normalization might be considered an alternative approach to correct the expression of urinary mRNA. In addition, our study underlines the importance of slit diaphragm proteins involved in calcium disequilibrium, such as TRPC6.

## 1. Introduction

The need to obtain valuable, sensitive, and specific information to recognize the signs of renal dysfunction and how it contributes to identifying glomerular disease and renal transplant (RTx) decline has recently led to new investigations. This research has involved both genomic, proteomic, and metabolomics studies, but unfortunately, at this time, definitive results have not been reached [1,2,3,4,5,6,7,8,9,10].

The research for the detection of novel markers in urine samples, for the description of renal damage, is of high interest. In this regard, the evaluation of urinary cell mRNA expression has proved to be a common and simple method to obtain information on fine gene transcriptional changes leading to renal disease [11,12,13].

A few studies performed in RTx patients have proven that the quantification of urinary podocyte mRNA markers may predict, also in the long-term [14], other than acute cellular rejection [14,15], subclinical glomerular alterations, prior to the progressive and irreversible RTx injury [16]. In all these studies, several strategies of normalization, to obtain and compare mRNA quantification, have been adopted [10,11,12,13,17,18,19,20]. In the present work, we have evaluated the urinary expression of a panel of specific podocyte genes, addressed to patients affected by renal disease with a clinical indication to RTx or native (N) renal biopsy (RBx).

We compared the urinary gene expression of each of these two groups of patients with those found in subjects with normal renal function. With the intent to dwell on functional aspects of podocyte biology leading to injury, we considered some of the crucial genes relevant for podocyte signaling pathways and the function of the glomerular filter applying an alternative normalization approach. Urinary podocytes mRNA quantification allowed us to identify further markers in patients with glomerular damage.

## 2. Materials and Methods

### 2.1. Controls

In the control group (CTRL) (*n* = 18) were considered subjects with normal renal function and with an absence of hematuria and proteinuria in urinalysis. The group was composed of 8 males and 10 females. All the control patients had a serum creatinine less than 1.1 mg/dl, and daily urinary protein excretion less than 0.150 g/24 h. The measure of urine creatinine was 1.29 ± 0.82 and 1.42 ± 0.55 g/L in women and men, respectively. Hematuria, proteinuria, and bacteriuria were assessed by dipstick urinalysis and, additionally, the presence of bacteria and yeasts was further excluded by microscopy inspection.

### 2.2. Patients

The cohort of patients considered in our study was composed of RTx (*n* = 20) and N (*n* = 18), (Table 1).

Both groups were equally represented among men and women. All the patients underwent RBx on clinical indication.

Briefly, the indications for RBx were: an increase of serum creatinine (sCr) >25% in respect to the basal level (A), an isolated appearance or increase of proteinuria >0.5 g/day (B), association of an increase of serum creatinine and increase of daily urinary protein excretion (A + B) and, in RTx, Polyomavirus (BK) virus replication in blood (>5000 copie/mL) (C).

All the patients were evaluated for renal-related blood and urinary parameters including urinary protein excretion (Prot-U), urinary creatinine excretion (UC), and measured glomerular filtration rate (mGFR).

Renal transplanted patients, according to the protocol present in our department, were treated with three immunosuppressive drugs: steroids, calcineurin inhibitors (cyclosporine/tacrolimus), and mycophenolate.

Patients who had undergone native renal biopsy were not taking any specific medication for their disease. In fact, the renal biopsy was performed before the initiation of the therapy.

The protocol was conducted according to the ethical principles of the Helsinki Convention, and each patient signed an informed consent. All data have been analyzed anonymously.

- RTx characteristics:

RTx ranged from an age of 24 to 75 years and had a mean time of RTx of 64 months (min 2–max 226 months). In those patients, the indications to RBx were distributed as A = 7 patients; B = 5 patients; A + B = 5 patients, and C = 3 patients. RBx were interpreted using the Banff’13 criteria [21,22]. Histological analysis did not report significant anomalies in the third RTx, whereas in the fourth and second ones, respectively, a rejection (T cell or antibody-mediated) and BKV nephropathy was found. Ten patients were affected by chronic transplant damage.

- N characteristics:

N ranged from an age of 20 to 72 years. In those patients, the indications to RBx were distributed as A = 2 patients; B = 11 patients; A + B = 5 patients. Histological analysis reported 7 subjects with lupus nephritis; 3 patients with membranous glomerulonephritis; 2 patients with renal amyloidosis, 3 patients with light chain deposition disease, 1 with diabetic nephropathy, and 1 with IgA nephropathy. In one case, alteration compatible with malignant hypertension was found whereas, in 1 patient, it was not possible to perform the biopsy for technical reasons.

### 2.3. Urine

About 40 mL of fresh urine were collected under fasting conditions between 7 a.m. and 10 a.m. the day of RBx and were stored in a sterile box at room temperature. The volume was divided in two aliquots, 30 mL to extract RNA and 10 mL to seed the cells in a culture flask. RNA extraction was performed within 4 h from harvesting to limit RNA degradation. Both volumes of urine were centrifuged at 2500× *g* for 15 min at 4 degrees, as previously described [23], to obtain a pellet containing cells originating from kidney and bladder but lacking in subcellular particles.

### 2.4. Biochemical Analysis

Biochemical analyses for the evaluation of kidney function were performed in the central laboratory of our institution and were measured the day of biopsy. mGFR was measured by means of 24 h urinary collection and by the measurement of creatinine clearance. The study was approved by the Institutional Review Board of Fondazione IRCCS Ca’ Granda Ospedale Maggiore Policlinico of Milan, protocol code 4759-1837/19 19 November 2019 and conducted according to the guidelines of the Declaration of Helsinki. Informed consent was obtained from all subjects involved in the study.

### 2.5. Human Immortalized Podocytes Culture

Human immortalized podocytes (hPODO) (University of Bristol, Bristol, UK) were used to check primers’ positivity and to compare the melting curve, as described in the supplementary section.

### 2.6. Immunofluorescence Staining

For immunofluorescence staining, the cells were fixed in cold 4% PFA and/or acetone as appropriate and permeabilized by 0.3% Triton X-100. The detailed process is reported in the supplementary section. To prevent unspecific binding, we used 5% bovine serum albumin for 30 min at room temperature. The following primary antibodies were applied: rabbit anti-podocin (*NPHS2*) (Sigma-Aldrich, Milan, Italy), guinea pig anti-nephrin (*NPHS1*) (Progen, Heidelberg, Germany), mouse anti-synaptopodin (*SYNPO*) (Progen, Heidelberg, Germany) 1:50, mouse anti-Wilms’ tumor suppressor 1 (*WT1*) (Sigma-Aldrich, Milan, Italy), goat anti-neurogenic differentiation factor one (*NEUROD*) (N-19) (SCBT, Heidelberg, Germany) 1:50, mouse anti-metabotropic glutamate receptor 1 channel (GMR1) (BD Pharmingen, BD Italia, Milano, Italy) 1:50, rabbit anti-transient receptor potential cation channel subfamily C, member 6 (*TRPC6*) (Abcam, Milano, Italy). After washing the appropriate secondary (Alexa Fluor) fluorescent 546- or 488-labelled antibodies (Invitrogen, Milano, Italy), the following were applied: goat anti-rabbit IgG, goat anti-mouse IgG, rabbit anti-goat IgG, goat anti-guinea pig, all diluted 1:70. DAPI (Sigma-Aldrich, Milan, Italy) nuclear staining was added with the secondary antibody. Direct staining with rhodamine-labelled phalloidin (Sigma-Aldrich, Milan, Italy) was used to analyse filamentous actin. Specificity of antibody labelling was demonstrated by the lack of staining after substituting proper control immunoglobulins for the primary antibodies. Slides were mounted with Fluorsave aqueous mounting medium (Merck, Milano, Italy). Images were acquired by a Zeiss Axioscope 40 FL microscope (Carl Zeiss SpA, Arese, Milan, Italy), equipped with a AxioCamMRc5 digital camera and immunofluorescence apparatus, and recorded by AxioVision software 4.8, or by a Zeiss AxioObserver microscope equipped with a high-resolution digital camera AxioCam and Apotome system (Carl Zeiss SpA, Arese, Milan, Italy) for structured illumination.

### 2.7. RNA Extraction

RNA was extracted by a urinary cells pellet. In addition, RNA obtained by normal renal tissue was used as a positive control of markers expression. Furthermore, RNA was extracted from a cellular line of hPODO (University of Bristol, Bristol, UK) for further use to compare positivity of podocyte markers, of melting curve, and for inter-assay variation. The tissue, cells, and urine pellets were washed with cold sterile PBS and RNA was extracted by Trizol (Thermo Fisher Scientific, Monza, Italy) reagent following the manufacturer’s instructions and, after DNAse treatment, diluted in 30µL of RNase-free water and stored at −80 °C.

### 2.8. Urinary Cells Culture

Cells were pelleted and seeded on Thermanox coverslip (Nunc, VWR Int., Milan, Italy) or in flasks pre-coated with collagen type IV, for cytochemical staining, morphological evaluation, and growth capacity, the day of urine collection, as detailed in the supplementary section.

### 2.9. RTqPCR

The RNA was transcribed into complementary DNA (cDNA) by iScript cDNA Synthesis Kit (BioRad, Segrate, Milan, Italy) obtained from various amounts of RNA. The amounts of 100 ng, 200 ng, and 250 ng RNA were preliminarily used to test the minimum quantity necessary to measure RNA expression of all selected markers in RNA, from each kind of starting material. After data comparison, 250 ng/mL were definitively used. mRNA from hPODO was used to assess inter-assay RTqPCR variability.

Prior to using cDNA for markers quantification by RTqPCR, SsoAdvanced PreAmp Supermix (BioRad, Hercules, CA 94547), with a primer mix of 25 different primers, 50 nM of each, was used to amplify the signal, as previously described [24]. For each sample, we prepared a pre-amplification reaction with 25 µL of SsoAdvanced PreAmp Supermix (BioRad, Hercules, CA 94547), 5 µL of primer mix, 20 µL of cDNA, in a final total volume of 50 µL. The reaction was used with the following thermal cycle condition: 3 min 95 °C, 15 s 95 °C, 4 min 58 °C, for 20 cycles. Pre-amplified cDNA was stored at −20 °C. Before use in RTqPCR, the pre-amplified solution was diluted 1:5 in distilled water.

Primers were chosen for their cell podocyte specificity or for function. Housekeeping genes were included in the mix: ribosomal protein L13 (*RPL13*), beta2 microglobulin (*beta2M*), glyceraldehyde 3 phosphate dehydrogenase (*GAPDH*), and *18sRNA*. We performed RTqPCR for a limited number of samples and for mRNA obtained by a human podocyte cell line, using the alternative housekeeping genes *RPL13*, *beta2M*, *GAPDH* and *18sRNA*, and two podocyte genes (*NPHS1* and *NPHS2*). We compared both their expression and repetition accuracy. We excluded the housekeeping genes *RPL13* and *beta2M* whose expressions were lower than that of *GAPDH*, and eliminated *18sRNA* because it was too high compared to that of *NPHS1* and *NPHS2*. Consequently, we decided to normalize podocyte genes in respect to *GAPDH*, as accepted in previous studies.

The more stable and expressed gene in comparable amounts was GAPDH, as also previously suggested [25], and was used to test.

Among markers contained in the primer mix, we considered and measured in this paper the following genes (Supplementary Table S1: primer sequences): *NPHS1*, *NPHS2*, *WT1*, *SYNPO*, *TRPC6*, *GRM1*, *NEUROD*.

The cases examined in this study are heterogeneous for both etiologies and clinical presentation. Patients in which renal biopsy was requested for diagnostic reasons have in common with transplant patients the presence of glomerular lesions. For this reason, we considered, in urinary cells, the expression of markers essential to verify the functionality of podocyte, particularly through calcium flow control. We concentrated on some of the specific podocyte molecules responsible for the maintenance of the slit diaphragm and on some ion channels involved in calcium homeostasis. *TRPC6* and *GRM1* activation or inactivation have been proposed to induce opposite mechanisms, leading to cell survival or death, as a form of regulation of homeostasis or apoptosis.

We selected genes characteristic of differentiated podocytes: *NPHS1*, *NPHS2*, *SYNPO*, and *WT1* [20,23,26]. In addition, as markers particularly involved in podocyte functionality, we considered and detected *TRPC6*, *GRM1*, and *NEUROD*.

RTqPCR was performed using iQ Sybr green Supermix (BioRad Hercules, CA 94547) and run with CFX Connect Real-Time System provided with CFX Manager Software for data acquisition and analysis (BioRad Hercules, CA 94547). Primers were purchased from Sigma.

At first, we calculated the expression levels by using the comparative 2-ΔΔCt method. The values were normalized to GAPDH and expressed relatively to CTRL group.

Furthermore, to obtain the best correction [17], we expressed the values as the ratio of UC.

However, in literature, it has been described as useful to represent the continuation of kidney injury and the related podocytes loss as the ratio of two cell specific markers [27].

Consequently and according to Trimarchi [24], we further expressed mRNA expression after *GAPDH* normalization as the ratio between two podocyte markers belonging to different cell compartments and further normalized to UC.

We chose *NPHS1* in representation of the slit diaphragm and *SYNPO* as molecules bound to actin cytoskeleton, and in addition, *WT1* as a nuclear marker.

### 2.10. Statistical Analysis

We expressed RTqPCR data as mean fold ± SE relative to CTRL, repeating the determination of each sample at least three times. Variables with skewed distribution were transformed in their base log_10_ + 1 to obtain normal distribution. To compare data, we used the unequal variance two-tailed Student’s *t*-test and, when necessary, used non parametric analyses (Mann–Whitney and Kruskal–Wallis tests). To ascertain correlation between groups, Pearson’s or Spearman’s nonparametric correlation, as appropriate, was performed. All statistical analyses were performed using GraphPad Prism software. The significance level of * *p* < 0.05, ** *p* < 0.01, or *** *p* < 0.001 was used.

## 3. Results

### 3.1. Cell Morphology and Growth

To ascertain if live cells were present in the urine, the urine pellet was maintained in culture; the presence of cells able to adhere was demonstrated by optical microscopy inspection day-by-day for the time of observation. After about two or three days, we noted cell adhesion and we could identify some podocytes [28,29] showing a large cytoplasm with first and secondary processes or long processes adhering to each other and with double nucleus in the same cytoplasm (Supplementary Figure S1: urinary cells culture).

We obtained podocyte cultures from both N and RTx urine, but not from CTRL, probably because of the low number along with the absence of proliferative capacity that characterized these terminally differentiated cells that were also described as senescent in healthy individuals.

The cells identified as podocytes did not proliferate over the monitored period. The characterization of podocytes was confirmed by immunocytochemistry staining. NPHS1 and NPHS2 proteins had different cellular distributions and, in some cells, were absent from the cell membrane and foot processes; *SYNPO* and *WT1* were well expressed (Figure S2: urinary podocytes characterization).

**Figure 1:**
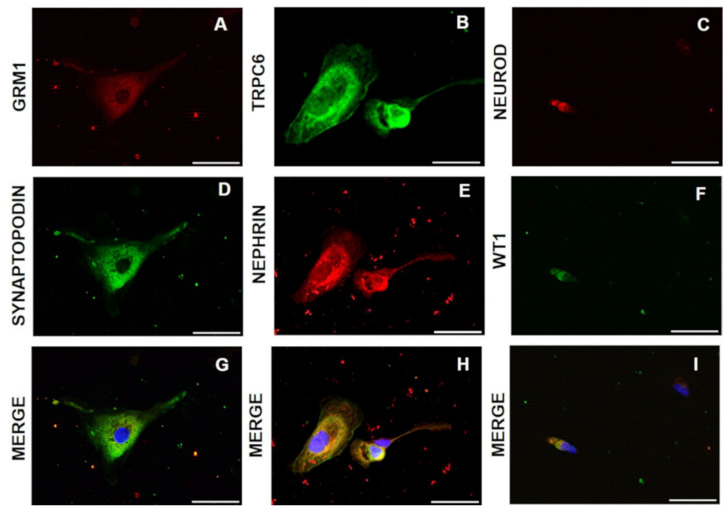
Staining of functional molecules in urinary podocytes. GRM1, TRPC6, and NEUROD (**A**–**C**) double staining with, respectively, synaptopodin, nephrin, and WT1 (**D**–**F**); Merge between A–C and D–F (**G**–**I**) cytoplasmic and nuclear podocyte markers were detected in urinary podocytes. DAPI Nuclear staining (blue) shows the absence of apoptosis. Scale bar is 100 µm.

Furthermore, we ascertained the presence of *TRPC6* and *GRM1* receptors and *NEUROD* transcription factor in podocytes for their functional roles (Figure 1).

**Figure 2:**
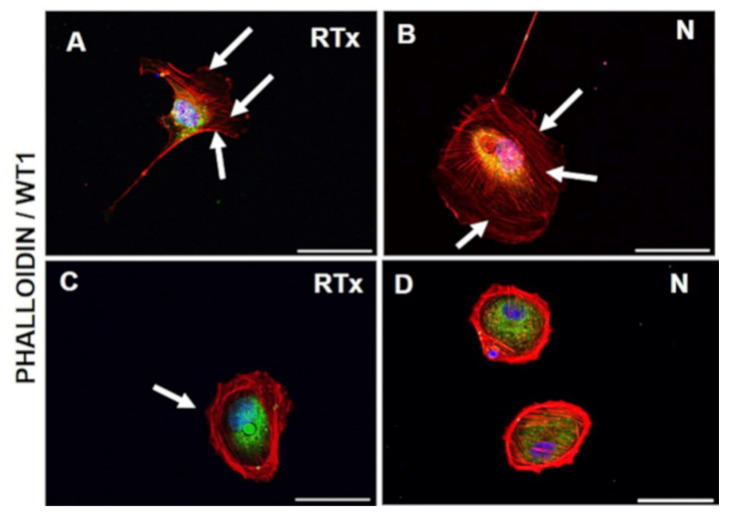
Cytoskeleton remodelling. Actin detected by phalloidin staining (red) shows different distribution in urinary cells of both N and RTx. WT1 nuclear staining identifies the podocytes (green); Podocyte with actin bundles aligned in the cytoplasm (**A**,**B**); other podocytes with altered appearances with round shapes, thickened actin bundles at the cell boundaries, and limited cytoplasmic stress fibers (**C**,**D**) DAPI (blue) nuclear staining shows the absence of apoptosis. Scale bar is 100 µm.

**Figure 3:**
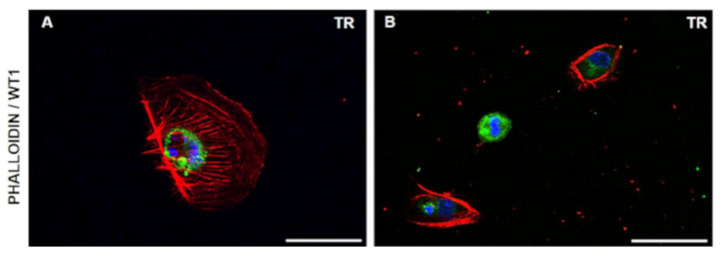
Actin distribution. Stress fibers (red) are visible in podocytes as dorsal, ventral, and transverse arcs (**A**), or disrupted and distributed along the edge of the cell and poorly expressed in the cytoplasm. (**B**) Double staining with nuclear podocyte marker WT1 (green) to demonstrate podocyte staining and DAPI (blue) nuclear marker. Scale bar is 100 µm.

The cells had different cytoskeletal features (Figure 2 and Figure 3). We noted that some podocytes had extended foot processes departing from cell bodies and were making contact with other cells; their actin bundles were aligned in the cytoplasm (Figure 2A,B) with stress fibers distributed with morphologies of various orientations (Figure 2A and Figure 3A). On the contrary, other podocytes had altered appearances with round shapes, thickened actin bundles at the cell boundaries, and limited cytoplasmic stress fibers (Figure 2C,D and Figure 3B).

### 3.2. Urinary mRNA Expression

We measured the mRNA expression level of *NPHS1*, *NPHS2*, *SYNPO*, *WT1*, *TRPC6*, *NEUROD*, and *GRM1* of urinary cells for their involvement in podocyte morphology, differentiation, and functional role.

**Figure 4:**
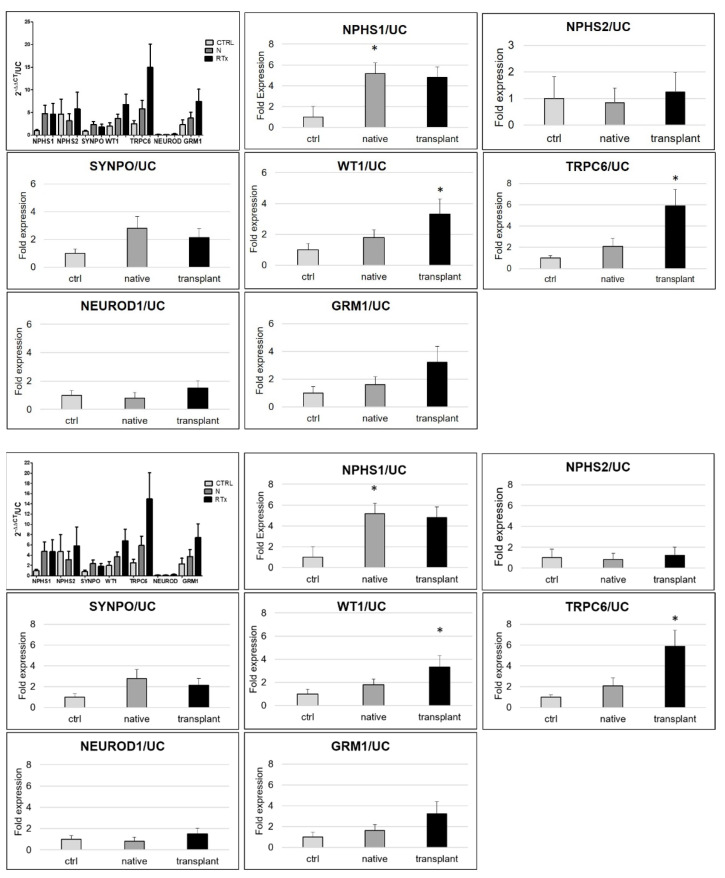
mRNA quantification of urinary cells. mRNA expression was measured in CTR, N, and RTx by RTqPCR, relative to *GAPDH* and corrected with UC. In the first image top left, the values are expressed as 2^−ΔΔct^/UC as mean ± standard error to display the amount of gene expression in each group of the sample. Differently, in the subsequent histograms, the results are expressed relative to the control group as mean fold expression ± standard error, to compare the groups. Significance * *p* < 0.05.

The mRNA quantification, relative to the housekeeping gene of the tested genes, demonstrated that *TRPC6*, *GRM1*, and *WT1* are the most expressed genes in urinary cells of N and RTx (Figure 4, first image top left)

In some cases, we detected low mRNA expression of some markers. *NPHS1* was low-expressed (>35 Ct) in 5/20 RTx. In the other 5/20 RTx, *NPHS1* was poorly expressed as was also the NPHS2 mRNA of the corresponding patients. This is not indicative of podocyte decrease or of mRNA degradation, because the other podocyte markers (WT1 and SYNPO) and GAPDH always resulted as being expressed in the overall cohort of RTx, N, and CTRL. These results, in addition, show that not only nephrin and podocin proteins, but also their mRNA, diminish in some patients.

mRNA expression of each marker, normalized by the *GAPDH* housekeeping gene and corrected for UC, was compared to CTRL group (Figure 4).

An overall significant increase of *NPHS1* in N (*p* = 0.05) and in RTx was found, as well as a strong tendency of *SYNPO* to increase in N and in RTx patients, presumably mirroring the increase of the number of podocytes; *WT1* and *TRPC6* resulted as occurring significantly higher than in CTRL in RTx by, respectively, 3.32 fold (*p* = 0.05) and 5.90 fold (*p* = 0.03). Notably, *TRPC6* and *NPHS1* occurred higher than in the CTRL, as well as in three RTx patients with no histological alterations. A less marked tendency to increase TRPC6 was observed in N. *GRM1* increase was measured but with an absence of significance. The expression of *NPHS2* and of *NEUROD* was comparable in all groups.

### 3.3. Correlation among the Different Markers after Normalization for Total Number of Cells

After the evaluation of the expression referred to total cells of the urinary pellet, we assessed the expression of the markers relative to surrogates of the number of urinary podocytes. For this purpose, we decided to use podocyte markers, although most of the podocyte genes under pathophysiologic condition could also be expressed by parietal epithelial cells transdifferentiated in podocytes [30,31].

At first, we verified if specific podocyte genes affected the mRNA expression of others, as we would expect from transcription factors (*WT1*, *NEUROD*) and from genes involved in the same pathway (*NPHS1*, *NPHS2, SYNPO*). To this end, we calculated the correlation among markers in the overall cohort but we found no significant correlation (Table S2), neither in N nor in RTx.

In this contest, we hypothesized a normalizing role by *WT1*, as an alternative and in combination with that of other genes, previously accepted to normalize podocytes expression in glomerular diseases.

### 3.4. mRNA Expression Related to Urinary Podocytes

**Figure 5:**
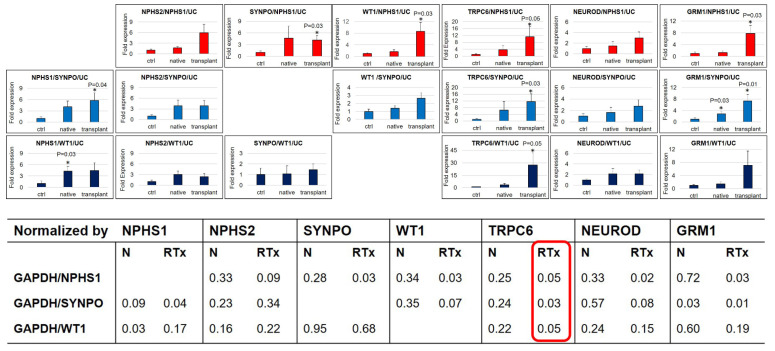
RTqPCR normalization for podocyte number. The fold expression of genes normalized with GAPDH was further normalized using the expression of, respectively, *NPHS1*, *SYNPO*, and *WT1*, followed by UC correction. Significance * *p* < 0.05. *p*-values (lower figure) obtained by comparison with the CTRL are calculated with two-tailed unpaired *t*-test.

We evaluated the mRNA expression relative to *NPHS1*, *SYNPO*, and *WT1*, as reported in Figure 5.

After correcting to *NPHS1*, we found an increase of all the considered markers in RTx, with a significant rise of *SYNPO*, *WT1, TRPC6*, and *GRM1*, and a strong tendency of *SYNPO* to increase expression in N.

Successively, as suggested in the literature [20], we performed *SYNPO* normalization; we observed the same trend, although less pronounced, of the previous ratio in all markers in RTx and a rising amount in N with an evident increase of *NPHS1* in N and RTx. We measured a significant difference of *NPHS1*, *TRPC6*, and *GRM1* in RTx relative to the CTRL.

Finally, we evaluated *WT1* normalization. Additionally, by this correction, we observed a general tendency to increase, with the maintenance of the significance only for *TRPC6* in RTx and of *NPHS1* in N. A correlation test of each gene between different normalization methods was performed and demonstrated a high relationship (data not shown).

## 4. Discussion

Multiple causes are responsible for renal injury and the search of early markers of dysfunction by a non-invasive method is an important unresolved matter.

In this contest, we aim to identify, by means of an observational study, the difference in mRNA expression of urinary cells among patients with RTx and patients with N and the CTRL

In consideration of our research experience, and differently from previous studies, we considered a larger group of glomerular markers involved in the function of slit diaphragm signaling. In fact, glomerular dysfunctions are crucial elements leading to glomerulopathies in native kidney, and responsible for the long-term RTx loss.

After the confirmation of the presence of podocytes in the urine samples, as commonly described in patients with different renal diseases [32,33,34], we determined the cell capacity to adhere and grow in vitro.

As reported in the literature, in healthy subjects, the cell senescence determines the loss of podocytes in urine and affects their survival in vitro [35]. Conversely, in patients with impaired renal function, podocytes detachment is due to still undetermined causes. According to the diversity in grow capacity observed in our samples, we also expected to detect a variability in RNA transcription.

First, we considered nephrin and podocin, the main actors of the functional cluster of the podocytes slit [36] whose alterations are involved in the genetic form of focal and segmental glomerulosclerosis (FSGS) [37,38] and are responsible for podocytopathies [31,39]. Their low expression indicates cellular suffering, probably reflecting an alteration in the structure and function of the podocytes’ ultrafiltration apparatus. In particular, the podocin expression level in micro dissected glomeruli was suggested as a diagnostic separator of glomerular disease [40]. By immunocytochemistry, we proved that nephrin and podocin were still detectable in RTx and N, although their distribution on cell membrane and foot processes was in some cases altered. In addition, to check the consequence of slit diaphragm signaling [41,42], we looked for changes in podocyte cytoskeletal morphology. According to these premises, we found different patterns of actin distribution, capable of undermining the cell stability.

At this point, we investigated the expression of genes particularly involved in the correct function of the slit diaphragms.

We proceeded first, in line with good practice, by estimating the gene expression relative to the housekeeping gene, as usual, to compare different groups with respect to the total cell number and to correct eventual RNA degradation; in addition, as reported in literature, and with the aim to obtain the most precise results, we corrected the findings also with UC to take into account urine concentration and differences in body composition [22].

By this method, in agreement with data present in the literature and relative to different renal pathologies [16,20,27,43,44], an increase in *NPHS1* expression, compared to CTRL, was found in N, and a relevant increase was measured in RTx. Differently, *NPHS2* was expressed equally in all groups. Among podocytes markers, we also evaluated the expression of *SYNPO* and *WT1*. An enhancement of both genes was found in N and in RTx, possibly reflecting the increase of urinary podocytes, consistent with the proceeding of glomerular injury.

The expression of the genes relative to proteins and receptors located in the slit diaphragm or in the nucleus, and strictly related to podocytes function, was also considered. The results point out high levels of *TRPC6* and of *GRM1* in all patients, but particularly in RTx, and no increase of *NEUROD*.

At this time, we faced the problem of the interpretation of these results to correctly compare the gene quantification in urine of different groups of patients. We wondered which of these results indicated a change in gene expression and which only a change in podocytes number. We considered it necessary to perform a correction of the gene expression in respect to the total number of podocytes. The choice of a reliable podocytes marker is actually a crucial point to resolve [7,25]. Not being able to directly count the number of podocytes, we tried to use alternative normalization approaches.

Previous studies suggested non-glomerular genes or specific podocytes markers to normalize gene expression with different assumptions [11,20,45]. In accordance with the previously reported literature and in relation to the stable expression of the gene in the entire cohort, we used in our samples the normalization with *SYNPO*, and with *NPHS1* for comparison, although the low expression of the latter may cause an overestimation of the ratio.

In addition, in consideration of the constant measurable and of its independent expression from *NPHS1, NPHS2*, and *SYNPO*, we assumed that *WT1* could be considered a performing surrogate of podocytes presence and quantity.

Finally, we examined the results in their entirety, offering a snapshot of podocyte mRNA expression in the different groups of patients, at the time of urine collection.

The data normalized with *NPHS1* showed a generalized increase of all markers, particularly in the RTx group; *SYNPO* normalization downgraded this result with a greater increase only in *NPHS1*, *TRPC6*, and *GRM1*. The results with the new normalizer *WT1* are more conservative, confirming the trend observed, but limiting the genes with a significant increase to *NPHS1* and *TRPC6*. Therefore, we suggest considering *WT1* correction of gene expression as a further, but not unique, appropriate means to compare gene expression of podocytes of different groups. *NPHS1* increasing in both N and RT is in agreement with previously reported studies [45].

In addition, normalized mRNA data show that *WT1* significantly increases in N and RTx after *NPHS1* normalization, suggesting an increase not only of the number of podocytes. The increase in gene expression of *WT1*, accompanied by the doubling of *NEUROD*, both transcription factors, contribute to keep the glomerular architecture mediating the mesenchymal-epithelial transition in case of dedifferentiation [46,47], and the cytoskeletal remodelling in case of injury [46]. However, *SYNPO* normalization did not confirm the result.

Our data demonstrate *TRPC6* overexpression. *TRPC6* is a mechanical sensor proposed to feel and respond to excessive filtrate flow, and to be inversely related to podocin gene activation [48,49,50]. As already known, ion channels play a role of primary importance in the regulation of calcium homeostasis [51,52,53]. In podocytes, *TRPC6* and *GRM1* participate to the modulation of the cell physiology, mediating the remodelling of the actin cytoskeleton and their imbalance, which determines glomerular damage and proteinuria in animal models and in humans [54,55,56,57,58,59]. *TRPC6* and *GRM1* trigger the Ca^2+^ entry, the former leading podocytes to motile phenotype with loss of stress fiber formation and cell rounding, a morphology that we observed in urinary podocytes by actin staining, and the latter mobilizing Ca^2+^ from intracellular store. The *GRM1* signal, mediated in neuronal cells by TRPC channels in postsynaptic membranes [46,60,61,62], plays an important role in synaptic plasticity [60] and is linked to excitotoxicity. We may suppose that the rise in *TRPC6* and *GRM1* reflects a disequilibrium in Ca^2+^ influx, partially responsible for the variation of the actin fibers’ distribution, as we observed by immunocytochemistry. Further investigations will be necessary to monitor if the *TRPC6* signaling pathway has been disturbed.

Obviously, the results of our study will be reconfirmed in a cohort of more patients, and the determination of urinary gene expression over time will provide more information about genes’ prognostic value and involvement in the outcome of renal disease. We hope that after future studies including more patients, the importance of the evaluation of these parameters will be also recognized in clinical practice as a standard diagnostic tool and assessment of therapeutic response. In our opinion, the urinary evaluation of these parameters might increase the knowledge derived from RBx or futuristically be a valid alternative to RBx, especially in those cases in which the procedure cannot be performed. Heretofore, their dosage can have a rationale only in clinical research.

On the other hand, our work is the first to explore functional neuron-like podocyte markers, especially in RTx patients. It could be seen as a strength, and in our opinion, it might represent a starting point for future studies.

## 5. Conclusions

According to our data, *WT1* normalization might be considered an alternative and careful approach to correct the expression of urinary mRNA. Our study suggests monitoring slit diaphragm gene expression, particularly of genes involved in calcium equilibrium, such as *TRPC6* and *GRM1*.

## Figures and Tables

**Table 1:** Clinical parameters and characteristics of analyzed patients. RBx: renal biopsy; M: Males; F: Females; Prot-U: urinary protein excretion; UC: urinary creatinine; sCr: serum creatinine; mGFR: measured glomerular filtration rate.

Characteristics	RTx	N
Number of patients	20	18
Age at RBx (years; mean ± SD)	55 ± 16	51 ± 14
Sex (*n*) (M/F)	9/11	8/10
Prot-U (g/L)	0.71 ± 0.83	2.44 ± 2.94
UC (g/L)	0.69 ± 0.39	0.73 ± 0.25
sCr (mg/dL)	1.91 ± 0.78	2.13 ± 2.50
mGFR (mL/min per 1.73 m^2^)	57 ± 28	80 ± 47

## Data Availability

If needed, data are available in anonymous form.

## Supplementary Materials

The following are available online at https://www.mdpi.com/article/10.3390/diagnostics11081499/s1, Figure S1. Urinary cells culture morphology, Figure S2. Urinary podocytes characterization, Table S1. Primer sequences, Table S2. Correlation among gene expression.
