# Supplementary material for: Urinary mRNA Expression of Glomerular Podocyte Markers in Glomerular Disease and Renal Transplant
Source: Diagnostics (Basel). 2021 Aug 20;11(8):1499. doi: 10.3390/diagnostics11081499 (PMC8392587; doi:10.3390/diagnostics11081499)
Supplement: Supplementary file 1 [file diagnostics-11-01499-s001.zip › diagnostics-1305250-supplementary.pdf]

Supplementary Material

Materials and Methods

Human Immortalized Podocytes Culture

hPODO were grown for propagation at 33 °C, 5% CO_2 in RPMI supplemented with 10% foetal bovine serum (FBS), 100 U/mL penicillin, 100 µg/mL streptomycin, and 2 mM L-glutamine (all reagents from Sigma-Aldrich, Milan, Italy). For differentiation, the cells were subsequently grown in the same medium with the addition of 5 µg/mL human apo-transferrin, 10^{-7} M hydrocortisone, 5 ng/mL sodium selenite, in culture flasks (Corning, Milan, Italy) pre-coated with collagen type IV and thermoshifted to 37 °C for 7 days.

Urinary Cells Culture

For the culture, the cells were washed with cold sterile PBS and diluted in DMEM: F12 medium supplemented with 10% FBS, 5 µg/mL human apo-transferrin, 10^{-7} M hydrocortisone, 5 ng/mL sodium selenite, 0.12 U/mL insulin, 100 U/mL penicillin, 100 µg/mL streptomycin, and 2 mM L-glutamine (all reagents from Sigma-Aldrich). Day-to-day cell adhesion was checked and images were taken by a Zeiss Axiovert 25 Microscope (Carl Zeiss SpA, Arese, Milan, Italy) for a total of 15 days. Samples with bacteriuria or other contaminations were excluded from the procedure. After adhesion, the cells plated on the chamber slide were frozen at –40°C for later characterization of cell types by immunofluorescence analysis.

RTqPCR

We considered and measured in this paper the following genes: *NPHS1, NPHS2, WT1, SYNPO, TRPC6, GRM1, NEUROD, GAPDH, RPL13, B2M*, and *18sRNA* (Supplementary Table S1).

Supplementary Table S1. Primer sequences. The following sequences of oligonucleotide primers were used.

GenBank	Gene	Forward primer sequence (5'-3')	Reverse primer sequence (5'-3')
NM_004646	NPHS1	CAACTGGGAGAGACTGGGAGAA	AATCTGACAACAAGACGGAGCA
NM_014625	NPHS2	AAGAGTAATATATTCCGACTGGGACAT	TGGTCACGATCTCATGAAAAGG
NM_007286	SYNPO	CCCAAGGTGACCCCGAAT	CTGCCGCCGCTTCTCA
NM_000378	WT1	GCATCTGAGACCAGTGAGAAA	TCCTGCTGTGCATCTGTAAG
NM_002500	NEUROD1	CACGCCAGTTTCACCATTTC	TGCAGCAGTAGTACCCAAAG
NM_004621	TRPC6	CTACTTTGAGGAGGGCAGAAC	CCCTGGAACAGCTCAGAAAT
NM_001278064	GRM1	GTATCCTACGCCTCTGTCATTC	GATCTCTGGCTTGTCTTGCT
NM_000977	RPL13	CCAGACACCAAGGTATGAGATG	GTCCATGAAGCAAGAACAATGG
NM_004048	B2M	CCAGCGTACTCCAAAGATTCA	TGGATGAAACCCAGACACATAG
NM_002046	GAPDH	GGTGTGAACCATGAGAAGTATGA	GAGTCCTTCCACGATACCAAAG
X03205	18SrRNA	GCCCGAAGCGTTTACTTTGA	TCCATTATTCCTAGCTGCGGTATC

Results

Cell Morphology and Growth

Urinary cells were maintained in culture and microscopy inspection was performed day-by-day for 14 days (Supplementary Figure S1).

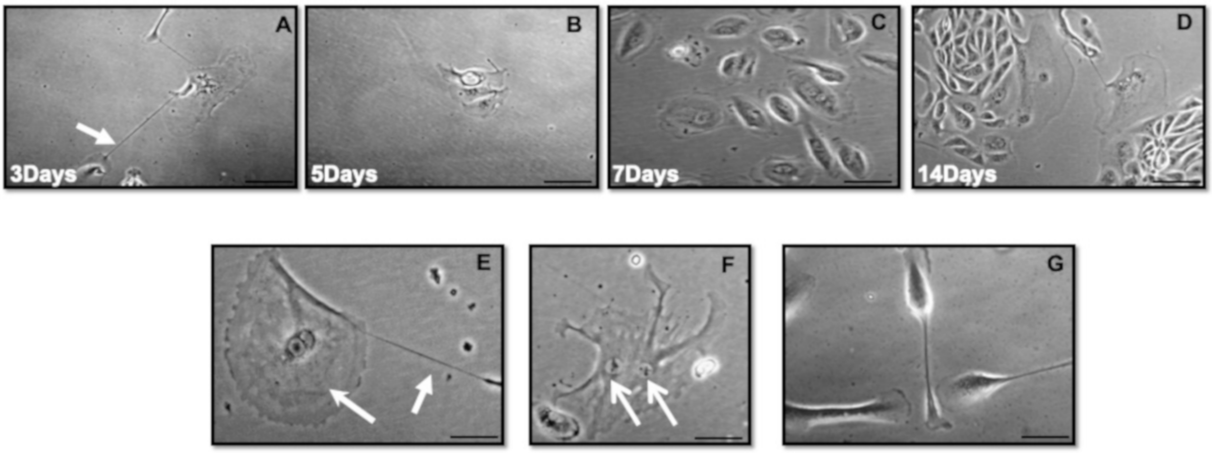

Supplementary Figure S1. Urinary cells culture morphology. Urinary cells were seeded in a flask for a culture in selective conditions for podocytes. After 2–3 days, cells of different kinds adhered or detached and were maintained in culture for 14 days. Light-inverted microscope images were acquired for 14 days (14d). Podocytes are recognizable by their processes (**A**–**E**), a large cytoskeleton (A,C,D,E), by a low duplication capacity (**B**) other than double nucleus (**C**–**F**), and processes trying to connect with those of other cells by synaptic-like structures (**G**). Scale bar for subfigures **A**–**F** is 100 μM; **G** 200 μM.

The characterization of podocytes was confirmed by immunocytochemistry staining (Supplementary Figure S2: Urinary podocytes characterization).

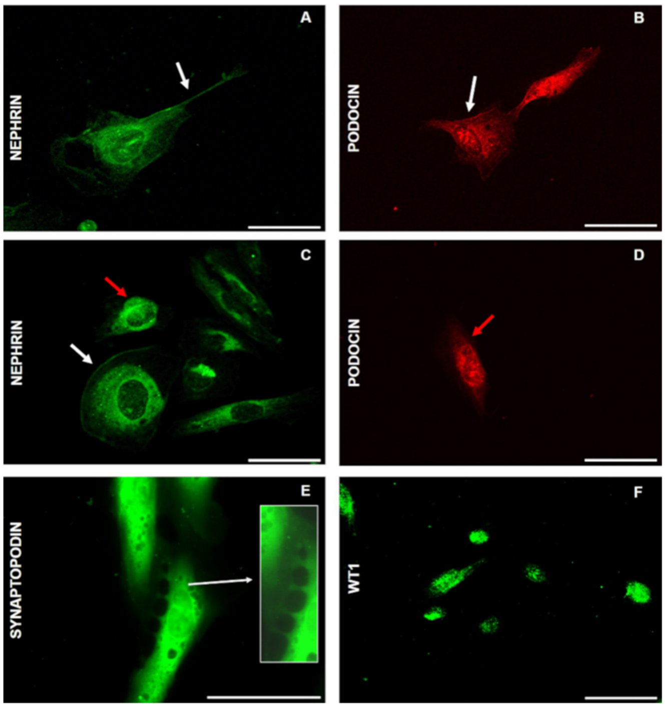

Supplementary Figure S2. Urinary podocytes characterization. Immunofluorescence staining was used to characterize podocytes. Podocytes expressed nephrin on cell membrane and foot processes (white arrow) (**A**–**C**) or into the cytoplasm (red arrow) (**C**); podocin was detected on the membrane (white arrow) (**B**) or in the cytoplasm (red arrow) (**D**); synaptopodin was found in cytoplasm and in the foot processes (**E**), and WT1 (**F**) in the nucleus, as expected. Scale bar is 100 μM.

Correlation among the Different Markers, after Normalization for Total Number of Cells

Pearson correlation among gene expression was performed in both RTx and N group after *GAPDH* and UC normalization after log + 1 transformation.

Supplemenatry Table S2. Correlation among gene expression. Pearson's correlation was performed after *GAPDH* and UC normalization.

RTx	NPHS1	NPHS2	WT1	SYNPO
NPHS1				
Pearson correlation		0.28	-0.10	-0.08
P value		0.25	0.70	0.77
NPHS2				
Pearson correlation	0.28		-0.02	0.17
p value	0.25		0.93	0.49
WT1				
Pearson correlation	-0.10	-0.02		0.21
p value	0.70	0.93		0.42
SYNPO				
Pearson correlation	-0.08	0.17	0.21	
p value	0.77	0.49	0.42	

N	NPHS1	NPHS2	WT1	SYNPO
NPHS1				
Pearson correlation		-0.29	0.16	0.25
p value		0.41	0.55	0.34
NPHS2				
Pearson correlation	0.41		0.34	0.39
p value	0.41		0.37	0.27
WT1				
Pearson correlation		0.34		0.73
p value		0.37		0.06
SYNPO				
Pearson correlation	0.25	0.39	0.73	
p value	0.34	0.27	0.06	
